# A Cross-Sectional Study of* Entamoeba histolytica/dispar/moshkovskii* Complex in Salvador, Bahia, Brazil

**DOI:** 10.1155/2019/7523670

**Published:** 2019-07-24

**Authors:** Neci M. Soares, Helen C. Azevedo, Flávia T. F. Pacheco, Joelma N. de Souza, Rodrigo P. Del-Rei, Márcia C. A. Teixeira, Fred L. N. Santos

**Affiliations:** ^1^Pharmacy College, Federal University of Bahia, UFBA, Salvador-BA 40170-115, Brazil; ^2^Faculty of Technology and Sciences of Bahia, FATEC-BA, Salvador-BA 40280-901, Brazil; ^3^Advanced Public Health Laboratory, Gonçalo Moniz Institute, FIOCRUZ-BA, Salvador-BA 40296-710, Brazil

## Abstract

Epidemiological studies on species-specific* Entamoeba* infections are scarce due to the morphological similarity of pathogenic* Entamoeba histolytica* and nonpathogenic* E. dispar* and* E. moshkovskii*. The diagnosis of* E. histolytica* is frequently based on coproantigen (*E. histolytica*-Gal/GalNAc lectin specific) detection by immunoassays. However, specific* E. histolytica*-lectin is not expressed in cysts, which are eliminated by asymptomatic individuals leading to false-negative results and an underestimation of amebiasis prevalence. Molecular techniques based on the amplification of parasite DNA have been shown to be a highly sensitive and specific method that allows the detection of different* Entamoeba* species. This study aimed to assess the frequency of the species from* E. histolytica/dispar/moshkovskii* complex by molecular and immunological techniques in individuals attended at a public health system in Salvador-Bahia, Brazil. A cross-sectional study involving 55,218 individuals was carried out. The diagnosis was based on microscopy revealing* E. histolytica/dispar/moshkovskii* complex. The species differentiation was performed by* E. histolytica*-specific antigen, serological evaluation and by molecular technique. The overall prevalence of* E. histolytica/dispar/moshkovskii* complex determined by microscopy was approximately 0.49% (273/55,218).* E. histolytica*-specific antigen detection and molecular characterization returned 100% negativity for* E. histolytica*. However, serological evaluation returned an 8.9% positivity (8/90). In the stool samples analysed by PCR, it was not possible to identify* E. histolytica* and* E. moshkovskii*, although circulating IgG anti-*E. histolytica* has been detected.

## 1. Introduction

*Entamoeba histolytica* is a common pathogenic protozoan widely distributed throughout the world. It is responsible for amoebic dysentery and amoebic liver abscess, resulting in human suffering and death. Amebiasis is significantly associated with food and drinking water supplies contaminated with human faeces and affects approximately 10% of the world's population, with 50,000-100,000 deaths reported annually, making this a significant cause of death due to protozoan parasites [1]. The* E. histolytica* prevalence is overestimated due to its epidemiological overlap with other morphologically identical species, i.e.,* Entamoeba dispar* and* Entamoeba moshkovskii*, currently composing the* E. histolytica/E. dispar/E. moshkovskii* complex [2–5]. Besides, the cysts of* Entamoeba hartmanni*, another nonpathogenic amoeba, even smaller in size (> 10 *μ*m), can be confused with* E. histolytica* cysts in the parasitological examination.

The prevalence of each species from this complex is not well characterized in many geographic regions. It is well-established that only* E. histolytica* leads to invasive disease in humans. Although some reports suggest a potential role of both* E. dispar* and* E. moshkovskii* in provoking disease in humans, they are still considered to be nonpathogenic and free-living amoebas, respectively [6–10]. Nevertheless, the differentiation of all* Entamoeba* species is substantial for preventing unnecessary and indiscriminate treatment with anti-amoebic chemotherapy, which could lead to drug resistance [11]. The World Health Organization advises that cases determined to have* E. histolytica* should be treated, whether or not clinical symptoms are present [12]. Additionally, control measures could be more efficiently applied in geographical areas with well-known epidemiological settings.

Regarding the low viability of trophozoites in diarrheal specimens and irregular faecal cyst excretion in asymptomatic hosts, the use of different diagnostic methods is required to increase the sensitivity of parasite identification in faecal samples. Immunoassays for coproantigen detection of specific* E. histolytica*-Gal/GalNAc lectin have been used as alternative methods for the diagnosis of amebiasis [13]. Molecular techniques based on the amplification of parasite DNA have been shown to be a highly sensitive and specific method that allows the detection of different* Entamoeba* species [14]. However, a negative result does not rule out the presence of the parasite due to interference from PCR inhibitors present in faeces that may hamper DNA amplification.

In Brazil, due to regional differences in sanitation and socioeconomic conditions,* E. histolytica/dispar/moshkovskii* complex distribution is irregular, with 2.5-11% in the South and Southeast, 19% in the North and the Amazon region, and approximately 10% in the Northeast and Central West [15]. In the city of Salvador, the capital of the Brazilian state of Bahia, the presence of the complex was shown in some studies conducted by our group with a prevalence of 5% and 3.2% in individuals attended at public [16] and the private health system respectively. Moreover, 15% (262/1,788) of positive stool samples for* E. histolytica/E. dispar* complex was analysed by PCR and no DNA amplified for* E. histolytica*. DNA amplified for* E. dispar* was observed in 86.6% of samples (227/262) [17]. However, in a previous study also conducted by our group, 4.6% (7/153) of hospitalized diarrheal children from Salvador tested positive for specific lectin of* E. histolytica*-Gal/GalNAc [18].

These findings further support evidence that* E. histolytica* may be infecting humans in Salvador. Given the importance of the actual prevalence of* E. histolytica*,* E. disp*ar, and* E. moshkovskii* infections, this study aimed to assess the frequency of each species by molecular and immunological techniques in individuals attended at the public health system in Salvador-Bahia, Brazil.

## 2. Materials and Methods

### 2.1. Ethical Considerations

Approval was granted by the Institutional Review Board (IRB) for Human Research at the Gonçalo Moniz Institute, Oswaldo Cruz Foundation (FIOCRUZ), Salvador, Bahia-Brazil, registration number 100/2006. All procedures were conducted in strict adherence to the principles laid out in the Declaration of Helsinki. Informed consent was obtained from participants who agreed to participate of the study. Samples were anonymously coded to protect each participant's identity.

### 2.2. Study Design and Population

**Figure 1:**
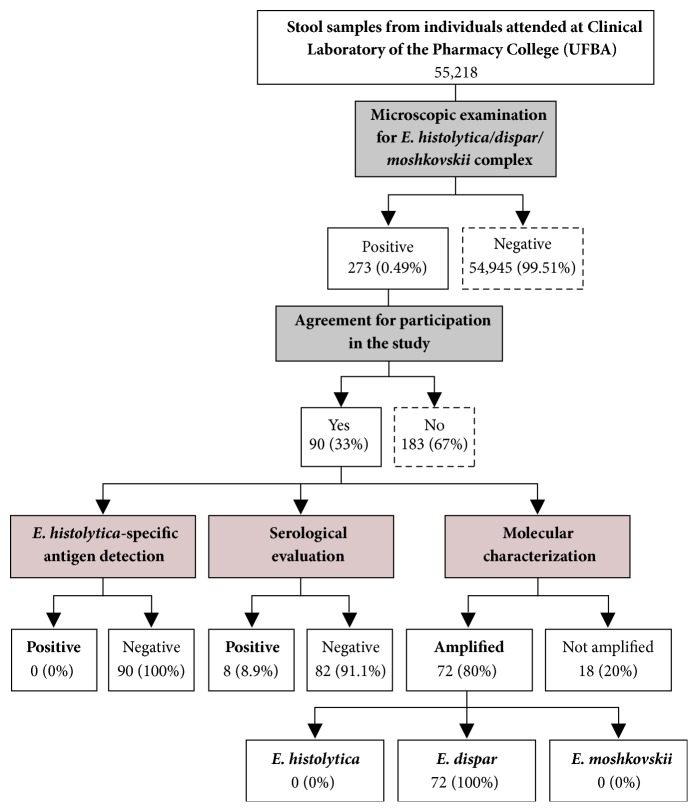
Flowchart summarizing the study design.

A cross-sectional study was performed from February 2010 to June 2014 involving 55,218 individuals who attended to the Clinical Laboratory of the Pharmacy College (LACTFAR; Federal University of Bahia). This population is mainly characterized by low-income individuals who are dependent upon the public health system. Stool examination was performed by laboratory staff. A flowchart illustrating the study design is provided in Figure 1.

### 2.3. Sample Selection

Two hundred and seventy-three patients diagnosed with amoeba cysts in their stool were contacted and invited to participate in the second phase of the study. A single fresh faecal specimen and 5 ml sample of blood for sera collection were obtained from 90 individuals who agreed to participate in the study. Coproantigen and IgG-specific antibodies detection and DNA amplification by PCR were used to diagnose the species of the complex.

### 2.4. Microscopic Examination

Approximately 3 g of faecal material was processed using the formalin-ethyl-acetate centrifugation method. Morphological analysis was conducted to detect the presence of tetranucleated cysts to confirm the diagnosis previously established by the spontaneous sedimentation technique. A 50 *μ*l portion of the concentrated sample was mixed with iodine, spotted on a glass slide and covered with a coverslip (24 × 24 mm). Slides were viewed at 40X magnification, and results were expressed as a number of cysts per gram of faeces.

### 2.5. Parasite Cell Culturing

*Entamoeba* trophozoites were obtained under xenic conditions in modified Pavlova's medium with bacterial flora, for coproantigen testing. Briefly, 1 gram of fresh stool was washed five times with distilled water, and 500 *μ*l of sediment was incubated at 37°C under 5% CO_2_ in 10 ml of Pavlova's medium in round-bottom threaded culture glass tubes (15 × 1.5 cm). Amoebas were maintained with thrice-weekly subcultures to ensure viability through the exponential growth phase. The transformation of cysts into trophozoites, as well as trophozoite development and viability, was monitored daily for five days.

### 2.6. *E. histolytica*-Specific Antigen Detection

Positive stool samples were preserved without fixative and stored at −20°C for posterior coproantigen testing (galactose adhesin) which was performed using the* E. histolytica* II assay (TechLab, Blacksburg, VA, USA). ELISA testing was carried out in accordance with manufacturer's directions. Absorbance at 450 nm was measured using a Bio-Rad Model 3550-UV Microplate Reader (Bio-Rad, CA, USA).

### 2.7. Serological Evaluation

The RIDASCREEN®* Entamoeba* test (R-Biopharm AG, Darmstadt, Germany) was used to detect specific anti-*Entamoeba histolytica* IgG in human sera. The method utilizes microtiter plates coated with purified antigens. Briefly, serum samples were loaded at 1:50 in sample diluent and incubated at RT for 15 min. Following incubation, the microplates were washed with washing buffer to remove any unbound antibodies. Protein A conjugate was added to each well, and the microplates were incubated for 15 min at RT. After five washes, the immune complexes were revealed by the addition of urea peroxidase substrate. After 15 min of incubation at RT in the dark, the reaction was stopped with stop solution, and the absorbance at 450 nm was measured using a Bio-Rad Model 3550-UV Microplate Reader (Bio-Rad, CA, USA). Cut-off value have established at OD = 0.3, in accordance with manufacturer's directions.

### 2.8. Genomic DNA Extraction

Positive stool samples were preserved without fixative and stored at −20°C until the time of DNA extraction. Genomic DNA was extracted directly from all samples using a QIAmp® DNA Mini Kit (QIAGEN, CA, USA) in accordance with the manufacturer's instructions. Briefly, 200 mg of stool sample was mixed with 1.4 ml ASL buffer in a microcentrifuge tube and vortexed until the sample was thoroughly homogenized. Following incubation at 70°C for 5 min, samples were centrifuged at 25,200 g for 1 min. InhibitEx tablets were subsequently added to the samples, followed by vortexing and centrifugation at 25,200 g for 3 min. Next, supernatants were transferred to new tubes, and then proteinase K was added. The tubes were reincubated at 70°C for 10 min. All samples were then mixed with ethanol and transferred to spin columns. After washing, DNA was eluted in 100 *μ*l elution buffer and immediately employed for PCR analysis.

### 2.9. Discrimination of* E. histolytica*,* E. dispar*, and* E. moshkovskii*

Nested multiplex PCR was carried out according to the protocol described elsewhere [19] using the 16S-like rRNA gene to discriminate between* E. histolytica*,* E. dispar*, and* E. moshkovskii*. The outer primer set, E-1 (5′-TAA GAT GCA CGA GAG CGA AA- 3′)/E-2 (5′-GTA CAA AGG GCA GGG ACG TA-3′), is specific to a shared fragment and was specifically designed for all three species. The inner primer pairs, EH-1 (5′-AAG CAT TGT TTC TAG ATC TGA G-3′)/EH-2 (5′-AAG AGG TCT AAC CGA AAT TAG-3′), ED-1 (5′-TCT AAT TCG ATT AGA ACT CT-3′)/ED-2 (5′-TCC CTA CCT ATT AGA CAT AGC-3′), and Mos-1 (5′-GAA CCA AGA GTT TCA CAA AC-3′)/Mos-2 (5′-CAA TAT AAG GCT TGG ATG AT-3′), are specific to 439-bp bracket for* E. histolytica*, 174-bp for* E. dispar*, and 553-bp for* E. moshkovskii*, respectively. Amplification was performed at a total volume of 25 *μ*l containing 0.3 *μ*M of each primer, 2.55 *μ*l 10X PCR buffer (200 mM Tris-HCl, pH 8.4, 500 mM KCl), 5.0 mM of each dNTP, 25 mM of MgCl2, 1.5 U Taq DNA polymerase (Invitrogen Life Technologies, CA, USA), and 2.5 *μ*l of DNA sample. An initial DNA amplification step was performed using the E-1/E-2 primer set in a MyCycler™ Thermal Cycler (Bio-Rad, CA, USA). The first cycle consisting of 2 min at 96°C was followed by 30 cycles of denaturation for 1 min at 92°C. Primers were annealed for 1 min at 56°C and extended for 1.5 min at 72°C. An additional extension step was performed at 72°C for 7 min. For nested amplification, 2.5 *μ*l of amplicon from the first reaction and primer sets EH-1/EH-2, ED-1/ED-2, and Mos-1/Mos-2 were used under identical conditions as those described above, with the exception of annealing temperature of 48°C. PCR products of tested samples and internal controls (DNA of* E. histolytica* and* E. dispar*) were analysed by gel electrophoresis. DNA fragments were separated on a 1.0% (w/v) agarose gel (Invitrogen Life Technologies, CA, USA) containing 0.5 *μ*g ethidium bromide/ml. Gels were photographed under ultraviolet illumination (Loccus Biotecnologia, SP, Brazil).

### 2.10. Data Analysis

Data were analysed using scatter plot graphing software (GraphPad Prism v.7, CA, USA). Descriptive statistics are presented as means or medians ± standard deviation, or percentages, to describe the characteristics of the studied population, including the prevalence of* E. histolytica*,* E. dispar*, and* E. moshkovskii*. The Shapiro-Wilk test was employed to assess data normality, and Levene's test was used to evaluate the homogeneity of variance. When these two assumptions were confirmed, ANOVA was used for sample comparisons; otherwise, Fisher's exact test was employed. All analyses were two-tailed and a p-value less than 5% was considered significant (p < 0.05).

## 3. Results

### 3.1. Analysis of Stool Samples

The routine parasitological analysis of 55,218 stool samples by microscopic examination identified* E. histolytica*-like cysts in 273 (~0.49%). The prevalence was significantly higher in adults (≥ 18 years). Similarly, there was a significant difference in the prevalence of infection between male and female subjects (Table 1). From the 273 positive individuals for the* E. histolytica/dispar/moshkovskii* complex, 90 (33%) agreed to participate in the survey (Figure 1).

### 3.2. *E. histolytica*-Specific Antigen Detection

Antigen detection by the* E. histolytica* II assay was employed to identify positive specimens for* E. histolytica*. All 90-stool samples tested were negative for* E. histolytica*, which was defined as an optical density value lower than 0.05 after subtraction of negative control value Additionally, it was verified if the negative antigen detection was due to the absence of trophozoites in the well-formed stool. Thirty-three samples were randomly selected and added to Pavlova's medium for growth of* Entamoeba* species. Incubation led to the growth of* E. histolytica/dispar/moshkovskii* in 28 (84.9%) cultures. Following the antigen detection of* E. histolytica* in the trophozoites transformed in culture, all specimens presented negative results.

### 3.3. Serological Evaluation

**Figure 2:**
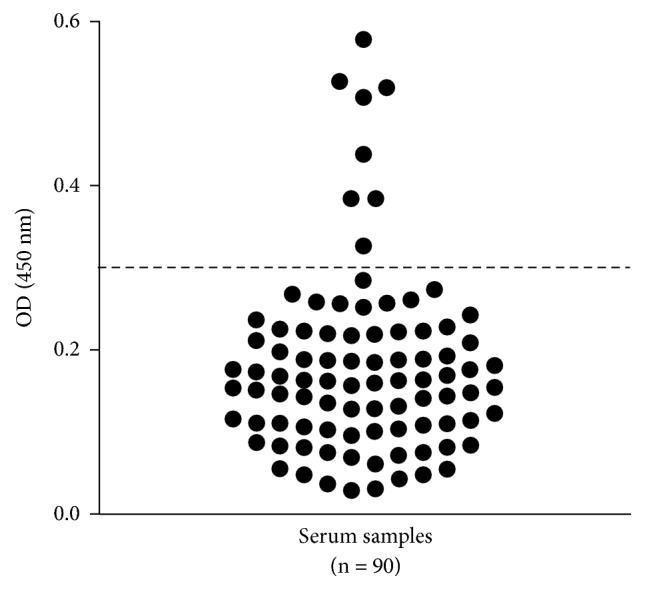
*E. histolytica*-specific IgG detection in 90 positive individuals for* E. histolytica/dispar/moshkovskii* complex attended at the Clinical Laboratory of Pharmacy College (Salvador, Brazil), from February 2010 to June 2014, by ELISA. The dotted line represents the cut off value of this test (OD = 0.3) and samples above this line are considered positive.

Out of 90 samples tested, eight (8.9%) were positive and 82 (90.1%) were negative by RIDASCREEN IgG antibody ELISA (Figure 2). Among the positive, no participant was suspected or had hepatic or extrahepatic* E. histolytica* infection, and all positive samples were positive for* E. dispar* under PCR analysis.

### 3.4. Molecular Characterization of* Entamoeba* spp.

**Figure 3:**
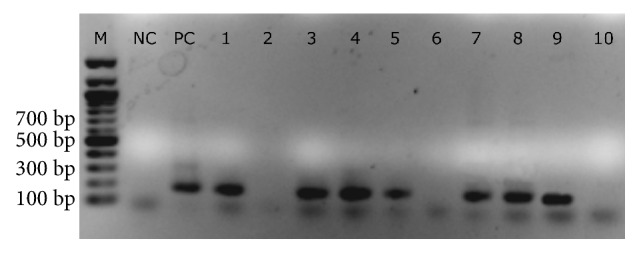
Agarose gel (1%) electrophoresis results for amplification of 72* E. histolytica/dispar/moshkovskii* complex positive samples by multiplex-PCR. M: molecular weight size marker (100 bp); Lane NC: negative control; Lane PC: positive control for* E. dispar*; Lanes 1, 3-5, and 7-9 depict the amplification results for* E. dispar*; Lanes 2, 6, and 10 depict negative results.

All 90 positive faeces samples for* E. histolytica/dispar/moshkovskii* complex were used in amplified PCR products. The 174-bp DNA fragment was successfully amplified in 72 samples (80%), indicating positivity for* E. dispar* (Figure 3). Multiplex PCR was capable of identifying the target in 42 samples directly from the DNA. However, 30 samples became positive only after successive dilutions (1:20 to 1:40). Eighteen samples remained negative even after diluted to 1:80 or more. The geometric mean of the counts of cysts per gram of faeces in amplified samples (390 ± 99) was higher than that found in non-amplified samples (196 ± 81; p = 0.02). No PCR products were detected for* E. histolytica* and* E. moshkovskii* species.

## 4. Discussion

Most of the epidemiological surveys on* E. histolytica* infection were conducted before the characterization of the* E. histolytica/dispar/moshkovskii* complex. Accordingly, new studies have been performed to discriminate these species and to establish the correct distribution of* E. histolytica* infection worldwide. In the present report, we employed immunological and molecular tools to determine the prevalence of* E. histolytica*,* E. dispar*, and* E. moshkovskii* infections among individuals attended at the Clinical Laboratory of Pharmacy College, in the city of Salvador (Bahia/Brazil). The prevalence rate of* E. histolytica/dispar/moshkovskii* complex based on faecal examination by optical microscopy was around 0.49%. This finding suggested a significant reduction in the number of cases compared to previous studies performed by our group. In fact, we demonstrated, in 2010, prevalence rates of 3.2% and 5.0% in samples from private and public clinical laboratories, respectively [16, 17]. This decrease could be explained in part by the positive impact of government programs, which have improved sanitation infrastructure, thus reducing the occurrence of intestinal parasites among residents of the Salvador Metropolitan Area [20]. Furthermore, the recent expansion of health education and health care services also may have contributed to the early identification of* E. histolytica* new cases and prompt treatment. Additionally, it appears that* E. histolytica* is more common in North and rare in other regions, including Salvador [17, 21, 22].

The PCR technique presented a sensitivity of 80% and specificity of 100%. Our data are in agreement with our previous report [17], in which we found a sensitivity value of 86.6%. In fact, 18 DNA samples did not amplify even after successive dilutions. It suggests either nondiagnosed* E. hartmanni* infections, which could be excluded by morphometric analysis and/or PCR [17, 23], or nonspecific substances present in the faecal sediment that inhibited DNA amplification [24]. Previous data from our group conducted in Salvador-Bahia showed that non-amplified DNA samples were not associated with* E. hartmanni* [17].* E. histolytica* coproantigen detection revealed negative results for all assayed samples, indicating no* E. histolytica* heterodimer galactose/N-acetyl-galactosamine (Gal/GalNac) lectin in faecal specimens. Significant variability in performance for* E. histolytica* antigen detection assays in both nonendemic [25, 26] and endemic areas [27, 28] has been reported. Conversely, a serological assay produced eight positive cases (8.9%), suggesting the previous exposure to the parasite. All the eight serologically positive samples rendered negative results for* E. histolytica* by PCR but positive for* E. dispar*. Antibodies remain detectable for years after successful treatment of* E. histolytica*, so it is difficult to distinguish between active and past infection reliably. Moreover,* E. histolytica* infection confirmed by lectin antigen can occur without antibody detection since the antigens search is more sensitive for* E. histolytica *diagnosis [29].

It is important to note that* E. dispar* has the same transmission path as other pathogenic protozoa, such as* E. histolytica*, indicating exposure to faecal contamination. Possibly, the production of IgG anti-*E. histolytica* is associated with immunological memory indicating the parasite circulation in Salvador, Bahia Brazil, in accordance with the previous data of our group [18]. Although some reports suggest a potential role of* E. dispar* in provoking intestinal and extra-intestinal symptoms in humans, its pathogenicity remains unclear [30]. The prevalence of* E. dispar* is 10 times higher than that of* E. histolytica* worldwide, and the attending physician must decide if treatment is necessary based more on clinical evidence [1].

## 5. Conclusions

In this study, the prevalence rate of* E. histolytica/dispar/moshkovskii* complex based on faecal examination by optical microscopy was around 0.49%. In the analysed samples by coproantigen and PCR, it was not possible to prove the presence of* E. histolytica* and* E. moshkovskii*. Only* E. dispar* was diagnosed by PCR, although the presence of circulating IgG anti-*E. histolytica* has been detected.

## Figures and Tables

**Table 1:** Distribution of *E. histolytica/dispar/moshkovskii* complex among individuals attended at the Clinical Laboratory of Pharmacy College (Salvador, Brazil), from February 2010 to June 2014 (n = 55,218).

Variables	No. examined	*E. histolyt*ica/*dispar*/*moshkovskii* complex	
		No. positive	%
*Age groups (years)*			
≤ 12	7,336	19	7.0
13-18	3,205	12	4.3
19-29	9,589	54	19.8
30-39	6,926	57	20.9
40-49	6,964	34	12.5
≥ 50	14,141	74	27.1
Missing data	7,057	23	8.4
Total	55,218	273	100
*Gender*			
Male	15,593	117	42.9
Female	32,435	144	52.7*∗*
Missing data	7,190	12	4.4
Total	55,218	273	100

*∗*p<0.05.

## Data Availability

The data supporting the conclusions of this article are provided within the article. The datasets generated and analysed during the current study are available from the corresponding author upon reasonable request.
